# Iodide of Potassium as an Antidote to Mercury

**Published:** 1853-10

**Authors:** J. W. Corson


*Iodide of Potassium as an Antidote to Mercury.* by J. W. Corson, M.D.—In common, doubtless with many others, I have for years been in the habit, especially in scrofulous constitutions, of always terminating a mercurial course by the protracted administration of the iodide of potassium, both as a remedy for the immediate effects of mercury, and as a prophylactic against its future injurious consequences. And I have gradually ventured to be far less apprehensive of the ill effects of the mercurials in cachectic habits when judiciously managed, than formerly.

These views came as quiet practical teachings from the bedside. But the peculiar mode of action by which the iodide of potassium neutralized the slow poison of mercury in the system, was to me, as doubtless to others, a mystery, till the recent clear and satisfactory experiments of M. Melsens, of Paris, explained the whole matter. The observations of M. Melsens were first embodied in a remarkably interesting paper, published not long since in the *Annales de Chimie et de Phisique*, and recently translated by Dr. Budd, of Bristol, and republished in the *British and Foreign Medico-Chururgical Review* for January, 1853. Those who are not already familiar with it we would refer to the original paper as a master-piece of medical observation, strengthening the patient deductions from the experience of the bedside, by the most rigid chemical analysis.

In the hope of directing more attention to these researches and somewhat confirming them, we have selected the above cases out of many others that might have been quoted. Some of them have the advantage of having been watched for several years, during which time they have exhibited no wandering pains, or undue sensitiveness to cold, and no cachectic appearances or other signs of injury from the constitutional effects of mercury. Four out of the five cases had legitimate evidence of scrofulous or tuberculous taints and were therfore, speccially liable to injury, and better proofs of the efficacy of the antidote. In the single exception, too, there was a chronic renal affection, and it is well known that in Bright's disease, and other chronic difficulties of the kidney, mercury is particularly apt to act at times with fearful violence. The suffering in this case from ptyalism, was most severe of all, and the prostration amounted almost to that of mercurial erethism.

Yet with these constitutional tendencies to hinder us in the administration, they all belonged to that desperate class of cases in which, from change of structure, or effusion of lymph or serum, life itself was immediately jeopardized, and apparently could only be saved by the resolvent effects of mercury. The simple enumeration of the cases would tell the practical physician that, when menacing life, he had, in duty to his patient, no other choice. They consist, it will be recollected, of extensive dropsy with hepatic congestion, threatening obstructive inflammation of the throat, immense pleuritic effusion, suffocative laryngitis, and severe puerperal peritonitis. In all these cases mercury appeared the *only remedy* capable of meeting the exigency. It succeeded most happily; and through the singular, and as we firmly believe efficient counteracting agency of the iodide of potassium, in a class of subjects most liable to injury, it left no sting behind. How the iodide of potassium acts in thus neutralizing the slow poison of mercury, the varied experiments of M. Melsens seems clearly to explain.

He lays it down as an admitted principle, proved by the well-known fact, that years afterward, persons who have once freely taken mercury find gold coins discolored by the mercurial in the perspiration of their bodies, and that mercury has been sometimes detected in the body after death; *that mercury as well as lead combines with the animal tissues*, and remains, so to speak, fixed in the system for years. Secondly, that in the body as well as out of the body, the iodide of potassium acts as a *powerful solvent to the compounds of mercury and lead; disengages them readily from the animal tissues*, and drains them off through the kidneys. Many ingenious chemical and clinical experiments are given to establish these propositions. M. Melsens first proved that the iodide of potassium passes off principally in the urine, by taking large quantities himself, and then analyzing the different secretions of the body. The fæces contained scarcely a trace, while the urine was loaded with it. It passes off through the kidneys with great rapidity. A person took 77 grains of the iodide of potassium, and in a few minutes the urine was charged with it. The compounds of mercury and lead, with the iodide of potassium, pass off by the kidneys in the same way.

An extraordinary cure of a looking-glass maker with severe mercurial paralysis, is given, in which the patient took the iodide of potassium in very large doses for several months, and repeatedly during this period the iodide of mercury was detected in the urine by chemical tests.

The great efficacy of the iodide of potassium as an antidote to the slow poison of lead and mercury, were proved by M. Melsens, by experiments upon several dogs, which were fed with the carbonate and sulphate of lead till paralyzed, emaciated, and nearly dead, and then in a short time restored to health and flesh by the administration of the iodide of potassium.

Three cases of severe lead paralysis among house-painters and workers in lead, were entirely cured, and a fourth greatly relieved by the same remedy. In five cases of mercurial paralysis and severe suffering among gilders and workers in quicksilver, the iodide of potassium in a few weeks, accomplished great relief, or a perfect cure.

It happened in some cases that the poison seemed to be liber ated so rapidly by the remedy that it was badly borne. Sometimes profuse re-salivation was the consequence. I had under my own care a gilder, aged 65, a few weeks since, suffering from mercurial tremors and paralysis, in whom eight grains of the iodide of potassium, three times a day, produced distressing ptyalism, and so added to his sufferings that he refused to continue the remedy.

Does the iodide of potassium ever salivate, except by *liberating* mercury?

We believe not. In the few cases in which salivation occurs from preparations of iodine, we think it will always be discovered that mercurials have at some previous time, in the patient's life, been taken.

From the experiments of M. Melsens, it appears that the iodide [of potassium when taken with mercurials, sometimes acts as a preventive to injury from the latter. We have for years been in the habit in strumous, syphilitic cases, of giving blue pill at night, and the iodide of potassium by day. Or we have *neutralized*, as we imagined the too severe effects of the prot. iodide of mercury, siny philtic and scrofulous throat affections, by combining with it the iodide of potassium. We have very lately witnessed excellent effects from this combination, in a case of tubercular syphiltic eruption.

Might not the exhibition of the iodide of potassium, in seasons of special exposure, be a protection to painters and workmen in lead, against lead colic, and paralysis?

It is curious to notice what immense quantities of the iodide of potassium may be often safely borne. M. Melsens took himself, for two months, from half a drachm to a drachm and a half per day, or more than two thousand grains in the whole period, without any inconvenience, except temporary coryza, and a few pimples, and with a decided increase of appetitite. One of the most severe cases of mercurial paralysis, related as cured by him, took 2,314 grains of the iodide of potassium, between the 21st of March and the 23d of June.

Of the cases of our own, one took five grains, three times a day, for eleven months, with the greatest benefit. M. Melsens reccommends, in cases of mercurial, or lead poisoning, to begin with fifteen grains in solution, three times a day, and to increase the dose as the patient will bear it.

Dr. Budd thinks such large doses require two conditions—*First*, to be given on an empty stomach; and *Secondly*, in a state of large dilution.

The cases we have narrated above seem to prove. that in milder forms where mercurial paralysis is not induced, and the system is not highly charged with the noxious mineral; smaller doses of the iodide of potassium, if continued sufficiently long, are highly efficacious.

In conclusion, these researches of M. Melsens explain, we think, why the iodide of potassium is so serviceable in certain broken-down syphilitic patients, in whom the quantity of mercurials previously taken, finally form an important element in their disease. We have not sufficiently tested the iodide of potassium in lead disease, to speak, as yet, with confidence from personal reference. We may, however, as a supplement to this paper, at a future period, report a few cases of lead paralysis, now under treatment, and hope, in the mean time, to promote the principal object of this paper by eliciting from the profession further observations on this important subject, and exciting a deeper interest in the original memoir, from which we have so freely quoted.—*New-York Journal of Medicine.*

